# Design and synthesis of an axially chiral platinum(II) complex and its CPL properties in PMMA matrix

**DOI:** 10.3762/bjoc.22.7

**Published:** 2026-01-15

**Authors:** Daiki Tauchi, Sota Ogura, Misa Sakura, Kazunori Tsubaki, Masashi Hasegawa

**Affiliations:** 1 Department of chemistry, Kitasato University, Kanagawa 252-0373, Japan; 2 Graduate School of Life and Environmental Science, Kyoto Prefectural University, Kyoto 606-8522, Japan

**Keywords:** axial chirality, chiral chemistry, circularly polarized luminescence (CPL), phosphorescence, platinum(II) complex

## Abstract

A pair of an axially chiral platinum(II) complex was synthesized via Sonogashira coupling and subsequent coordination of a pincer ligand to a precursor. The complex exhibited a broad absorption band ranging from 250 to 550 nm in the UV–vis spectrum, with TD-DFT calculations indicating mixed ligand-to-ligand charge transfer (LL’CT) and metal-to-ligand charge transfer (MLCT) character. Photoluminescence measurements in CH_2_Cl_2_ solution revealed dual emission peaks at 427 nm and 596 nm, with a quantum yield of 3%. In PMMA matrix, the emission peaks were blue-shifted to 408 nm and 558 nm, and the quantum yield slightly increased to 4%. CD spectra showed distinct Cotton effects in the MLCT region, and CPL signals were observed only in the PMMA matrices, with a dissymmetry factor |*g*_lum_| = 0.4 × 10^−3^. These results demonstrate that axial chirality of the binaphthyl moiety governs the three-dimensional chiral arrangement of two platinum(II) chromophore units, leading to the chiroptical properties in the MLCT region through exciton coupling under restricted molecular motion.

## Introduction

Luminescent materials based on metal complexes have been extensively studied due to their high phosphorescence efficiency, making them promising candidates for applications in organic light-emitting diodes (OLEDs) [1–5], sensors [6–9], and bioimaging materials [10–11]. Among these materials, pincer-type platinum(II) complexes have attracted particular attention, owing to the structural robustness imparted by tridentate chelating ligands and their excellent luminescent properties [12–17]. These complexes have been widely investigated, not only for practical application but also from the perspective of fundamental chemistry.

In recent years, the design and synthesis of luminescent pincer-type platinum(II) metal complexes incorporating chirality, as well as the study of their physicochemical properties, have gained significant interest [18–22]. Such efforts aim to explore the development of materials exhibiting circularly polarized luminescence (CPL). However, most reported chiral pincer-type platinum(II) complexes utilize point chirality as the chiral element. For instance, Zhong and co-workers reported a chiral platinum(II) complex bearing a leucine-derived pendant group, which exhibited aggregation-enhanced red phosphorescence and CPL properties in the solid states [20], while Yam and co-workers also reported pincer-type platinum(II) complexes containing chiral amine moieties, and investigated their aggregation behavior and chiroptical properties [22]. In both cases, significant CPL signals were observed only in the aggregated state, because the introduction of chirality through point chirality does not induce a significant electronic effect in the monomeric state; instead, the observed chiroptical activity originates from the chiral spatial arrangement of several molecules in the aggregated state. To expand the scope of chirality in metal complex chemistry with pincer ligands and to facilitate the design of next-generation CPL-active materials, alternative chiral motifs beyond point chirality are highly desirable.

**Figure 1:**
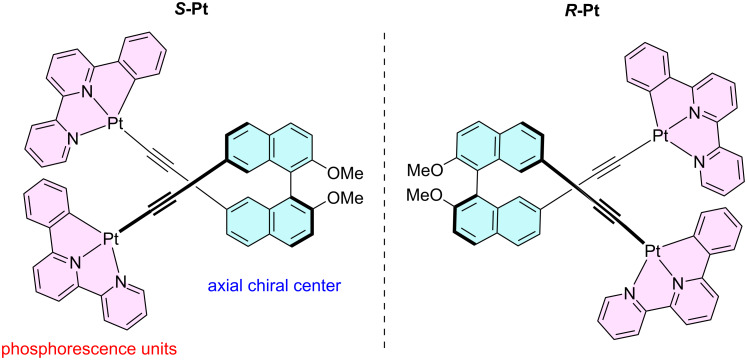
Molecular design for axially chiral platinum(II) complex ***S*/*R*-Pt** based on a pincer ligand.

Herein, we report the design and synthesis of a chiral ligand featuring an axially chiral binaphthyl backbone and its employment in the construction of novel types of a chiral platinum(II) complex with pincer ligand (Figure 1). We investigated the spectroscopic properties of the resulting complex, including its chiroptical behavior, through a combination of experimental measurements and density functional theory (DFT) calculations. The experimental UV–vis spectrum was in good agreement with the DFT calculations, revealing that the electronic transitions originate from both ligand-to-ligand charge transfer (LL’CT) and metal-to-ligand charge transfer (MLCT). Furthermore, chiroptical measurements by circular dichroism (CD) and CPL spectroscopy indicated that the axial chirality of the binaphthyl moiety governs the relative orientation of the Pt(II)-based chromophore pair, generating MLCT-band CD and CPL signals through exciton coupling in both the ground and excited states. While no CPL was observed in solution, distinct CPL properties observed in the PMMA matrices. This behavior is attributed to the effect of the axial chirality, which becomes more effective upon suppression of molecular motion within the polymer environment.

## Results and Discussion

### Synthesis

**Scheme 1:**
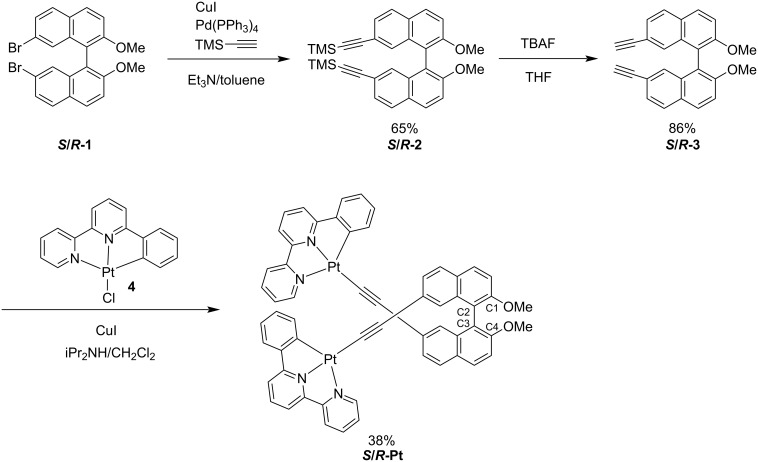
Synthesis of the binaphthyl-based ligand and the platinum(II) complex. Yields indicated correspond to the *S*-isomer.

Each enantiomer of the target platinum(II) complex was synthesized separately. The synthesis of the chiral backbone ***S*/*R*-3** was prepared in two steps starting from a chiral binaphthyl derivative [23]. Compound ***S*/*R*-2** was obtained by using a Sonogashira coupling reaction of ***S*/*R*-1** with trimethtylsilylacetylene in toluene (Scheme 1). Subsequent deprotection of the trimethylsilyl (TMS) groups with tetrabutylammonium fluoride (TBAF) afforded the desired ligand ***S*/*R*-3**. The preparation of the target platinum(II) complex was performed by using a similar procedure of other platinum(II) complexes with pincer ligand moieties [12]. Thus, the reaction of ***S*/*R*-3** with platinum(II) precursor **4** in the presence of CuI and diisopropylamine afforded the target platinum(II) complex ***S*/*R*-Pt** in moderate yield. The identification of the complex ***S*/*R*-Pt** were carried out with ^1^H and ^13^C NMR spectroscopy, as well as high-resolution mass spectrometry (HRMS). Due to its low solubility, single crystals suitable for X-ray crystallographic analysis could not be obtained.

### Spectroscopic analysis and DFT calculations

**Figure 2:**
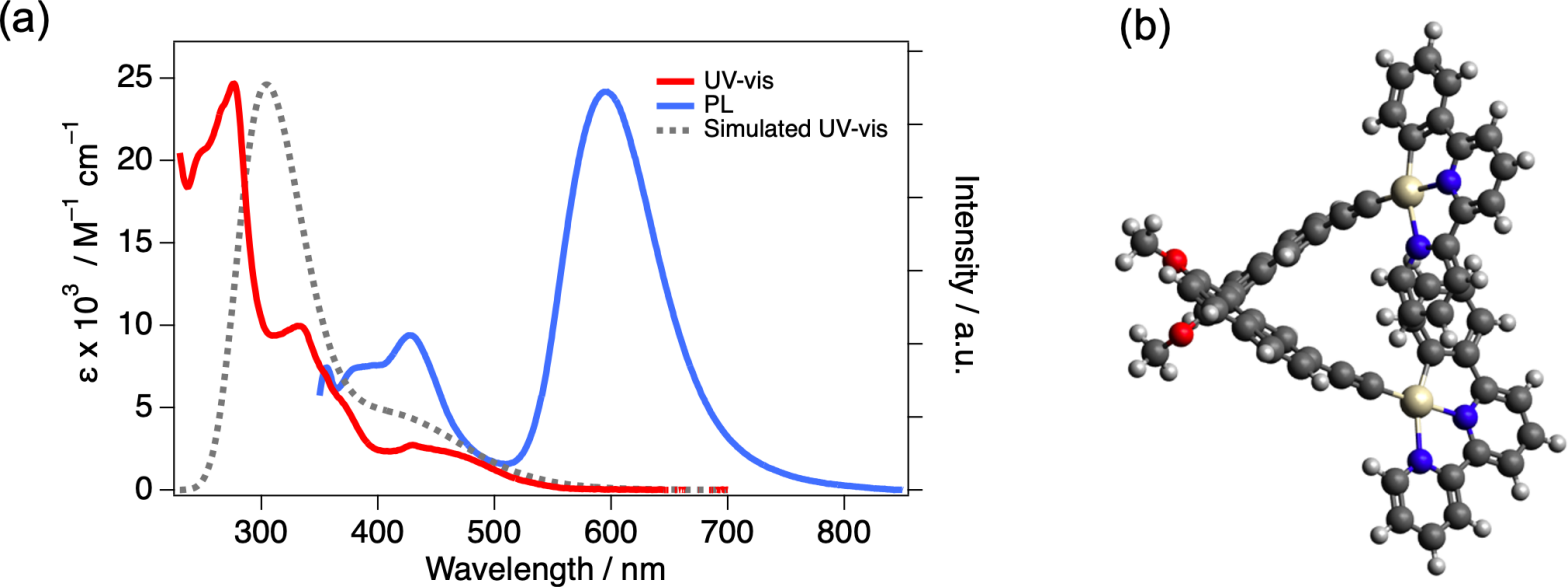
(a) UV–vis and PL spectra (λ_ex_ = 300 nm) in 1.0 × 10^−5^ M dichloromethane solution, the gray dotted line shows a simulated UV–vis spectrum by TD-DFT calculation. (b) Optimized structure of ***R*-Pt** by DFT calculation.

To elucidate the electronic structure of the platinum(II) complex, UV–vis absorption spectroscopy was performed in dilute solution of dichloromethane (1.0 × 10^−5^ M) (Figure 2a). The absorption spectrum displayed wide range absorption character from 250 to 700 nm with maxima at 430, 331, and 276 nm. To gain deeper insight into the nature of these electronic transitions, density functional theory (DFT) calculations were carried out. The calculations were performed at the CAM-B3LYP/6-31G+(d,p) level, with the LANL2DZ basis set applied to the Pt atoms. The optimized structure of ***R*-Pt** is shown in Figure 2b. The dihedral angle of the binaphthyl moiety (∠C1–C2–C3–C4) was 71°, which is smaller than the 80–100° range typically reported for the most stable conformation of binaphthyl groups [24]. In contrast, the dihedral angles between the binaphthyl unit and the coordination plane were 54° and 52°, respectively. The UV–vis spectral simulation based on electronic transition obtained from time-dependent (TD)-DFT qualitatively reproduced the observed spectra. The details of the electronic transition were attached in Supporting Information File 1. The broad absorption band in the long-wavelength region was assigned to a MLCT transition, while the absorption bands in the shorter wavelength region were attributed to a mixture of LL’CT and MLCT transitions. These electronic characteristics are similar to those reported for other chiral platinum(II) complexes [25–27].

The photoluminescence (PL) spectrum of the platinum(II) complex was measured in dichloromethane solution (1.0 × 10^−5^ M), revealing two emission bands centered at approximately 427 nm and 596 nm (Figure 2). The corresponding emission decays were well fitted by a three-exponential function, yielding fluorescence components of 3.6 ns (62%), 8.0 ns (20%), and 0.39 ns (18%) at 427 nm, and phosphorescence components of 0.11 μs (64%), 69 ns (35%), and 6.3 ns (1%) at 596 nm, respectively. The photoluminescence quantum yield (PLQY) in dichloromethane was measured to be 3%. The relatively low PLQY is likely attributed to non-radiative deactivation pathways associated with rotational motions of the axially chiral moiety and the ethynyl-linked coordination sites. Therefore, we prepared a PMMA polymer matrix containing 1 wt % of ***S*/*R*-Pt** to investigate the emission behavior under conditions where molecular motion was suppressed.

**Figure 3:**
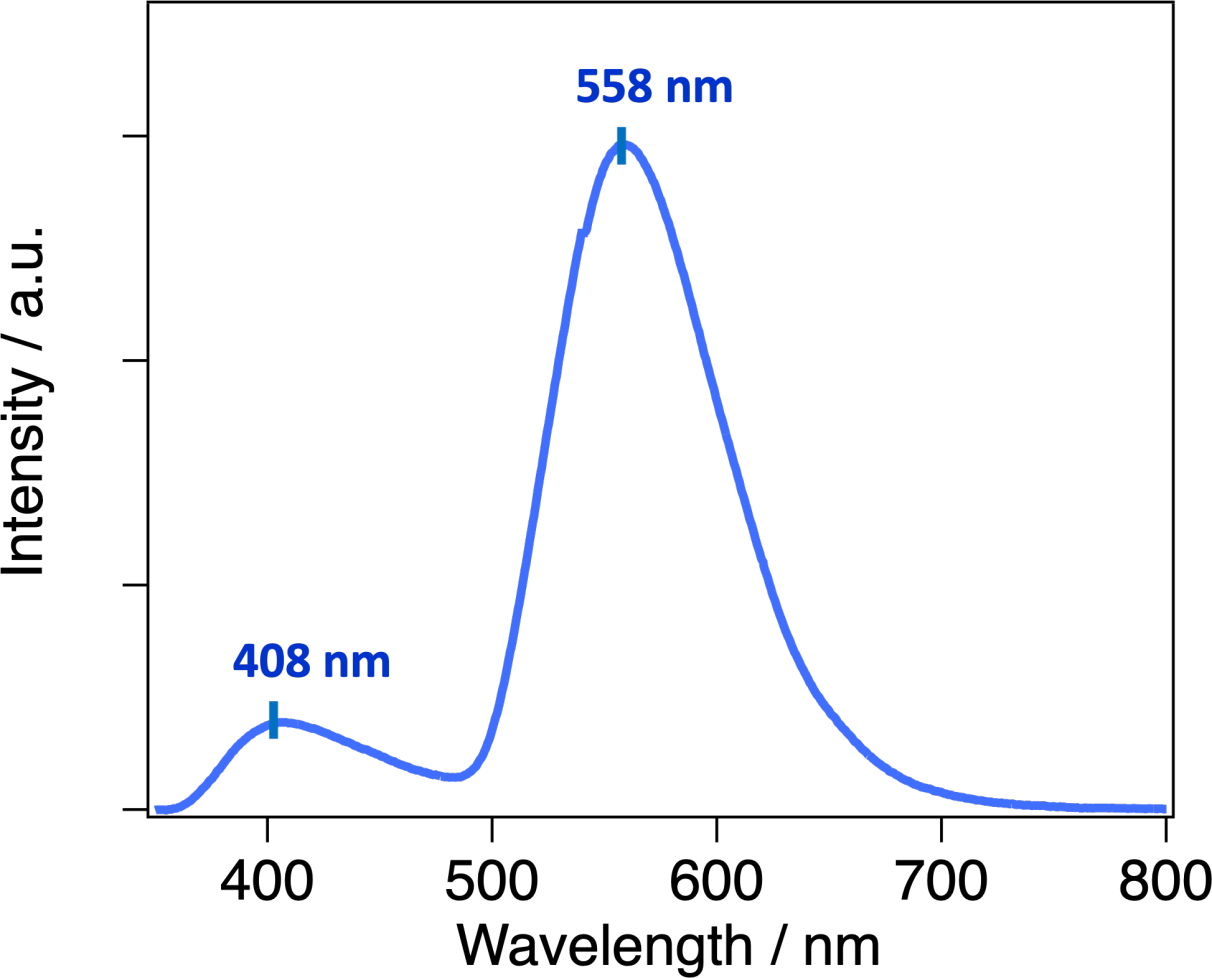
Emission spectrum of 1 wt % PMMA matrix (***R*-Pt**) (λ_ex_ = 300 nm).

The PMMA matrix was obtained by dissolving ***S***/***R*-Pt** and PMMA in chloroform under heating at 40 °C, casting the solution onto a glass substrate, and allowing it to dry slowly under ambient conditions. The emission spectrum of the PMMA matrix exhibited two emission components, similar to those observed in solution (Figure 3). Compared to the solution phase, the longer-wavelength emission peak (558 nm) was blue-shifted by 38 nm, and the shorter-wavelength emission peak (408 nm) was blue-shifted by 19 nm. These blue shifts are attributed to the hydrophobic environment in the PMMA matrix. In such low‐polarity surroundings, the excited state is less stabilized, resulting in a higher excited‐state energy and thus a blue‐shifted emission. The PLQY in the PMMA matrix was 4%, comparable to that observed in solution. In the film, the emission lifetimes could be fitted with double- or triple-exponential functions, resulting in fluorescence components of 0.49 ns (50%) and 0.48 ns (50%) at 408 nm, and phosphorescence components of 0.25 μs (76%), 0.024 μs (11%), and 2.8 ns (10%) at 558 nm, consistent with similar excited-state dynamics in both environments.

**Figure 4:**
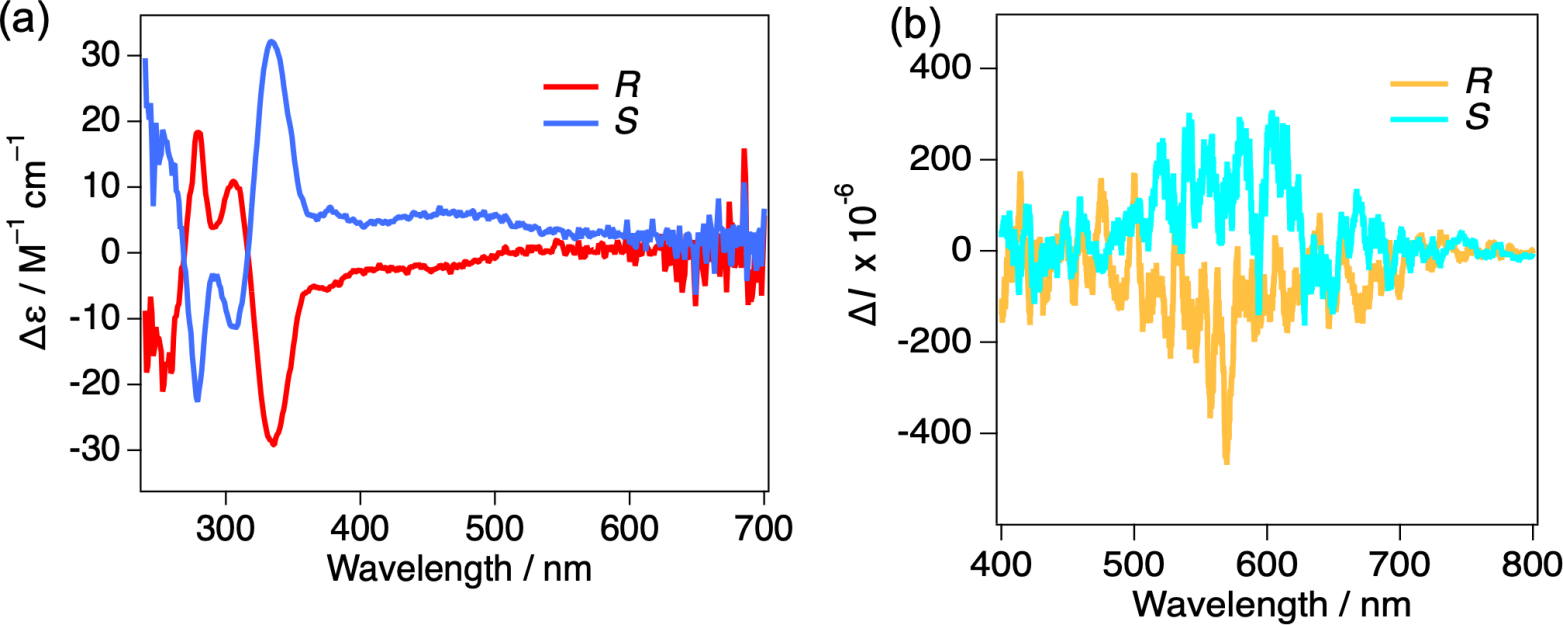
(a) CD spectra of ***S*/*R*-Pt** in 1.0 × 10^−5^ M dichloromethane solution. (b) CPL spectra of 1 wt % PMMA film (λ_ex_ = 300 nm).

To investigate the chiroptical properties of the complex, CD spectra were recorded in 1.0 × 10^–5^ M dichloromethane solution (Figure 4a). The CD spectra of both enantiomers showed clear mirror-image signals, and the experimental results were in good agreement with the simulated spectra obtained from DFT calculations (see Supporting Information File 1). The first Cotton effect attributed by MLCT transition was observed around 400 to 500 nm, indicating the axial chirality of the binaphthyl groups reflect the CD signals. It assumes circularly polarized phosphorescence (CPP) properties by platinum(II) centered emission. The TD-DFT calculation also reproduces the CD spectrum, showing the same sign in the first Cotton effect (*g*_abs_ = 1.1 × 10^−3^ at 404 nm). The corresponding *g*_abs_ chart is included in Supporting Information File 1. The CPL measurements were conducted to further elucidate the chiral emissive properties of the platinum(II) complex. The dichloromethane solution of the complex showed no detectable CPL signal, this is likely due to averaging of the transition dipole moments caused by intramolecular motions, leading to a cancellation of dipole moment or potentially weak magnetic dipole moment. In contrast, 1 wt % PMMA matrices exhibited distinct CPL activity, with the CPL signal showing the same sign as the first Cotton effect in the CD spectrum (Figure 4b). The dissymmetry factor is defined as follows: *g*_lum_ = 2Δ*I*/*I* = 2(*I*_L_ – *I*_R_)/(*I*_L_ + *I*_R_), where *I*_L_ and *I*_R_ denote the luminescence intensities of left- and right-handed circularly polarized luminescence, respectively. The measured dissymmetry factor |*g*_lum_| was 0.4 × 10^−3^ (at 558 nm), which is within the typical range for organic small-molecule systems [28–30], indicating the restriction of molecular motions in PMMA matrices induced CPL properties. The *g*_lum_ value was significantly smaller than the *g*_abs_ value in solution, which is considered to originate from structural flexibility in the excited state [28].

## Conclusion

In conclusion, we synthesized a novel pincer-type platinum(II) complex by incorporating an axially chiral binaphthyl ligand through an acetylene linker. Spectroscopic and theoretical analyses revealed that the axial chirality of the binaphthyl moiety imposes a chiral arrangement on the pair of platinum(II)-based chromophores, giving rise to MLCT-band CD and CPL through exciton coupling rather than through chirality induction at the platinum(II) center. The complex exhibited dual emission and distinct CPL activity in PMMA matrices, with a dissymmetry factor |*g*_lum_| of 0.4 × 10^−3^, highlighting the role of restricted molecular motion in enhancing chiroptical properties. These findings underscore the potential of axial chirality as a design principle for developing chiral phosphorescent materials and open avenues for further exploration of chiral metal complexes in optoelectronic applications.

## Experimental

### General information

All reagents and solvents were of commercial reagent grade and used without further purification. Dichloromethane for spectroscopy was purchased from FUJIFILM Wako Pure Chemical Corporation. All compounds were identified by ^1^H , ^13^C NMR and ESI-MS. ^1^H and ^13^C NMR spectra were recorded on a Bruker Avance III 400 or JEOL JNM-ECZ600R spectrometer at 25 °C in chloroform-*d*_1_ or DMSO-*d*_6_. ^1^H NMR chemical shifts are expressed in parts per million (δ) relative to trimethylsilane (TMS) as a reference. Mass spectra were obtained with a Thermo Scientific, Exactive Plus Orbitrap mass spectrometer for electrospray ionization (ESI). IR spectra were measured using a FT/IR-4600 spectrometer with KBr pellets. UV–vis absorption spectra of CH_2_Cl_2_ solutions (1.0 × 10^−5^ M) were recorded with a JASCO V-560 UV–vis spectrometer. Photoluminescence spectra of CH_2_Cl_2_ solutions (1.0 × 10^−5^ M) and 1 wt % PMMA matrices were acquired using a JASCO FP-8550 spectrometer at room temperature, at excitation wavelength of 300 nm (both solution and PMMA matrix). CPL spectra of CH_2_Cl_2_ solutions (1.0 × 10^−5^ M) and 1 wt % PMMA matrices were measured with a JASCO CPL-300 spectrofluoropolarimeter at room temperature, at a scattering angle of 0° upon excitation with unpolarized, monochromated incident light. Absolute PLQYs of solutions and powder samples were determined using a JASCO FP-8550 spectrometer with an integrating sphere (JASCO ILF-533, diameter 96 mm) at an excitation wavelength of 300 nm. Fluorescence lifetimes were measured for solutions and powder samples with a HORIBA DeltaFlex spectrometer with a 370 nm LED light source for excitation. For the lifetime measurements of solution, a conventional 1 cm quartz cell was used. Optical rotation values were measured with a JASCO P-1030 polarimeter using the sodium D line (λ = 589 nm). All calculations were performed by using the Gaussian 16 rev C.02 program package. Density functional theory (DFT) calculations were performed at the level of CAM-B3LYP/LANL2DZ for Pt atoms. The optimized equilibrium structures were confirmed by normal coordinate analyses, with no imaginary frequency found.

### Synthesis of axially chiral ligand and Pt(II) complex

#### Synthesis of ***S*/*R*-2**

The following describes the procedure for the *S*-enantiomer. Under an argon atmosphere, a mixture of ***S*-1** (282 mg, 0.597 mmol), CuI (32 mg, 0.17 mmol), Pd(PPh_3_)_4_ (112 mg, 0.0962 mmol) and trimethylsilylacetylene (0.40 mL, 2.9 mmol) was added degassed solution of Et_3_N (2.6 mL, 19 mmol) and toluene (5.2 mL) by argon bubbling. The reaction mixture was refluxed at 90 °C for 21 hours. After cooling to room temperature, the reaction mixture was filtered through Celite. The Celite was thoroughly washed with dichloromethane, and the combined filtrate was washed three times with saturated aqueous NH_4_Cl and brine. The organic layer was dried over anhydrous sodium sulfate. The crude product was purified by silica gel column chromatography using a 1:1 mixture of dichloromethane and hexane as the eluent. The eluent was evaporated to give the off-white solid ***S*-2** (197 mg, 65%).

Data for ***S*-2**: Off white solid; mp 103 °C (dec); HRMS (orbitrap) *m*/*z*: [M + H]^+^ calcd. for C_32_H_34_O_2_Si_2_, 529.1990; found, 529.1990; ^1^H NMR (400 MHz, CDCl_3_) δ 7.94 (d, *J* = 9.2 Hz, 2H), 7.79 (d, *J* = 8.4 Hz, 2H), 7.45 (d, *J* = 8.8 Hz, 2H), 7.36 (dd, *J* = 1.6, 8.4 Hz, 2H), 7.202–7.198 (m, 2H), 3.74 (s, 6H), 0.17 (s, 18H); ^13^C NMR (100 MHz, CDCl_3_) δ 155.5, 133.7, 129.4, 128.9, 128.7, 128, 126.7, 121, 119.1, 114.7, 106.1, 94.2, 56.6, 0.05; IR (KBr) ν_max_: 3058, 1774, 1409, 1191, 1152, 969, 698, 553, 431 cm^−1^. The *R*-isomer was synthesized using a similar procedure to that of the *S*-isomer, except that compound ***R*-1** (300 mg, 0.52 mmol), trimethylsilylacetylene (0.30 mL, 2.2 mmol), CuI (32 mg, 0.17 mmol), Pd(PPh_3_)_4_ (112 mg, 0.096 mmol), Et_3_N (2.3 mL) in toluene (4.5 mL) were used. The yield was 78%.

#### Synthesis of ***S*/*R*-3**

Under an argon atmosphere. ***S*-2** (197 mg, 0.389 mmol) in tetrahydrofuran (1.7 mL) was treated with a dropwise addition of 1 M TBAF solution (16.7 mL, 16.7mmol), and the resulting solution was stirred at room temperature for 2 hours. After the addition of water, the mixture was extracted with dichloromethane three times, and the combined organic layer was washed with saturated brine. The organic layer was dried over anhydrous sodium sulfate, after drying, the solvent was removed under reduced pressure. The crude product was purified by silica gel column chromatography using a 1:1 mixture of dichloromethane and hexane as the eluent. The eluent was evaporated to give the white crystalline solid ***S*-3** (121 mg, 86%).

Data for ***S*-3**: White crystal; mp 160 °C (dec); HRMS (orbitrap) *m*/*z*: [M]^+^ calcd. for C_26_H_18_O_2_, 362.1301; found, 362.1299; ^1^H NMR (400 MHz, CDCl_3_) δ 7.96 (d, *J* = 8.8 Hz, 2H), 7.81 (d, *J* = 8.4 Hz, 2H), 7.47 (d, *J* = 8.8 Hz, 2H), 7.37 (dd, *J* = 1.6, 8.4 Hz, 2H), 7.26 (s, 2H), 3.76 (s, 6H), 2.95 (s, 2H); ^13^C NMR (100 MHz, CDCl_3_) δ 155.5, 133.5, 129.5, 129.4, 128.8, 128.2, 126.4, 119.9, 118.9, 114.9, 84.6, 77.1 56.7; IR(KBr) ν_max_: 3238, 2938, 2839, 2457, 1352, 1320, 1171, 1152, 961, 882, 597, 530, 432 cm^−1^.

The *R*-isomer was synthesized using a similar procedure to that of the *S*-isomer, except that compound ***R*-2** (208 mg, 0.41 mmol) and TBAF (1.7 mL) in THF (17 mL) were used. The yield was 81%.

#### Synthesis of ***S*/*R*-Pt**

The synthesis of platinum(II) complex describe the *R*-enantiomer. Under an argon atmosphere, a mixture of ***R*-3** (16 mg, 0.043 mmol), **4** (39 mg, 0.084 mmol), and CuI (2.4 mg, 0.013 mmol ) were dissolved in degassed dichloromethane (10 mL) and diisopropylamine (10 mL). The reaction mixture was refluxed for 28 hours. After cooling to room temperature, water was added, and the mixture was extracted three times with dichloromethane and saturated aqueous NH_4_Cl. The organic layers were washed with saturated brine, then dried over anhydrous sodium sulfate. After drying the solvent was removed under reduced pressure. The crude product was then purified by silica gel column chromatography using a 9:1 mixture of dichloromethane and methanol as the eluent. The eluate was evaporated, and the resulting solid was washed with methanol and dried under vacuum to afford the orange solid of ***R*-Pt** (24 mg, 47%).

Data for ***R*-Pt**: Orange solid; mp 280 °C (dec); HRMS (orbitrap) *m*/*z*: [M + H]^+^ calcd. for C_58_H_38_N_4_O_2_Pt_2_, 1212.2343; found, 1212.2342; ^1^H NMR (400 MHz, DMSO-*d*_6_) δ 8.81 (d, *J* = 5.2 Hz, 2H), 8.41 (d, *J* = 8.0 Hz, 2H), 8.25 (t, *J* = 8.0 Hz, 2H), 8.14 (d, *J* = 8.0 Hz, 2H), 8.00–8.07 (m, 4H), 7.93 (d, *J* = 8.0 Hz, 2H), 7.85 (d, *J* = 8.4 Hz, 2H), 7.67–7.75 (m, 2H), 7.50–7.57 (m, 6H), 7.32 (dd, *J* = 1.2 and 8.4 Hz, 2H), 7.20–6.95 (m, 6H), 3.77 (s, 6H); ^13^C NMR (100 MHz, DMSO-*d*_6_) δ 164.1, 157.6, 155.0, 154.0, 150.8, 147.1, 142.2, 140.1, 137.6, 133.7, 130.7, 129.1, 128.5, 127.8, 127.2, 126.8, 126.7, 126.1, 125.0, 124.1, 123.6, 119.3, 119.2, 117.9, 113.4, 110.1, 109.5, 106.2, 56.3; IR(KBr) ν_max_: 3448, 3061, 2923, 2846, 2371, 2345, 2091, 1610, 1499, 1458, 1255, 1092, 880, 835, 757, 477 cm^−1^. 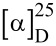 = –409 (*c* 0.0123, CH_2_Cl_2_).

The ***S*-Pt** complex was synthesized using a similar procedure to that of the ***R*-Pt** complex, except that compound ***S*-3** (20 mg, 0.055 mmol), **4** (51 mg, 0.11 mmol), diisopropylamine (10 mL) in CH_2_Cl_2_ (10 mL) were used. The yield was 25 mg, 38%.

## Supporting Information

File 1 Spectroscopic details and theoretical calculations.

## Data Availability

Data generated and analyzed during this study is available from the corresponding author upon reasonable request.
