# Supplementary material for: Design and synthesis of an axially chiral platinum(II) complex and its CPL properties in PMMA matrix
Source: Beilstein J Org Chem. 2026 Jan 15;22:143–50. doi: 10.3762/bjoc.22.7 (PMC12816981; doi:10.3762/bjoc.22.7)
Supplement: File 1 — Spectroscopic details and theoretical calculations. [file Beilstein_J_Org_Chem-22-143-s001.pdf]

BEILSTEIN JOURNAL OF ORGANIC CHEMISTRY

Supporting Information

for

Design and synthesis of an axially chiral platinum(II) complex and its CPL properties in PMMA matrix

Daiki Tauchi, Sota Ogura, Misa Sakura, Kazunori Tsubaki and Masashi Hasegawa

Beilstein J. Org. Chem. **2026,** *22,* 143–150. doi:10.3762/bjoc.22.7

Spectroscopic details and theoretical calculations

License and Terms: This is a supporting information file under the terms of the Creative Commons Attribution License (https://creativecommons.org/licenses/by/4.0). Please note that the reuse, redistribution and reproduction in particular requires that the author(s) and source are credited and that individual graphics may be subject to special legal provisions.
The license is subject to the *Beilstein Journal of Organic Chemistry* terms and conditions: (https://www.beilstein-journals.org/bjoc/terms)

Table of contents

1. **Spectroscopic details**
2. **Theoretical calculations**

1. Spectroscopic details

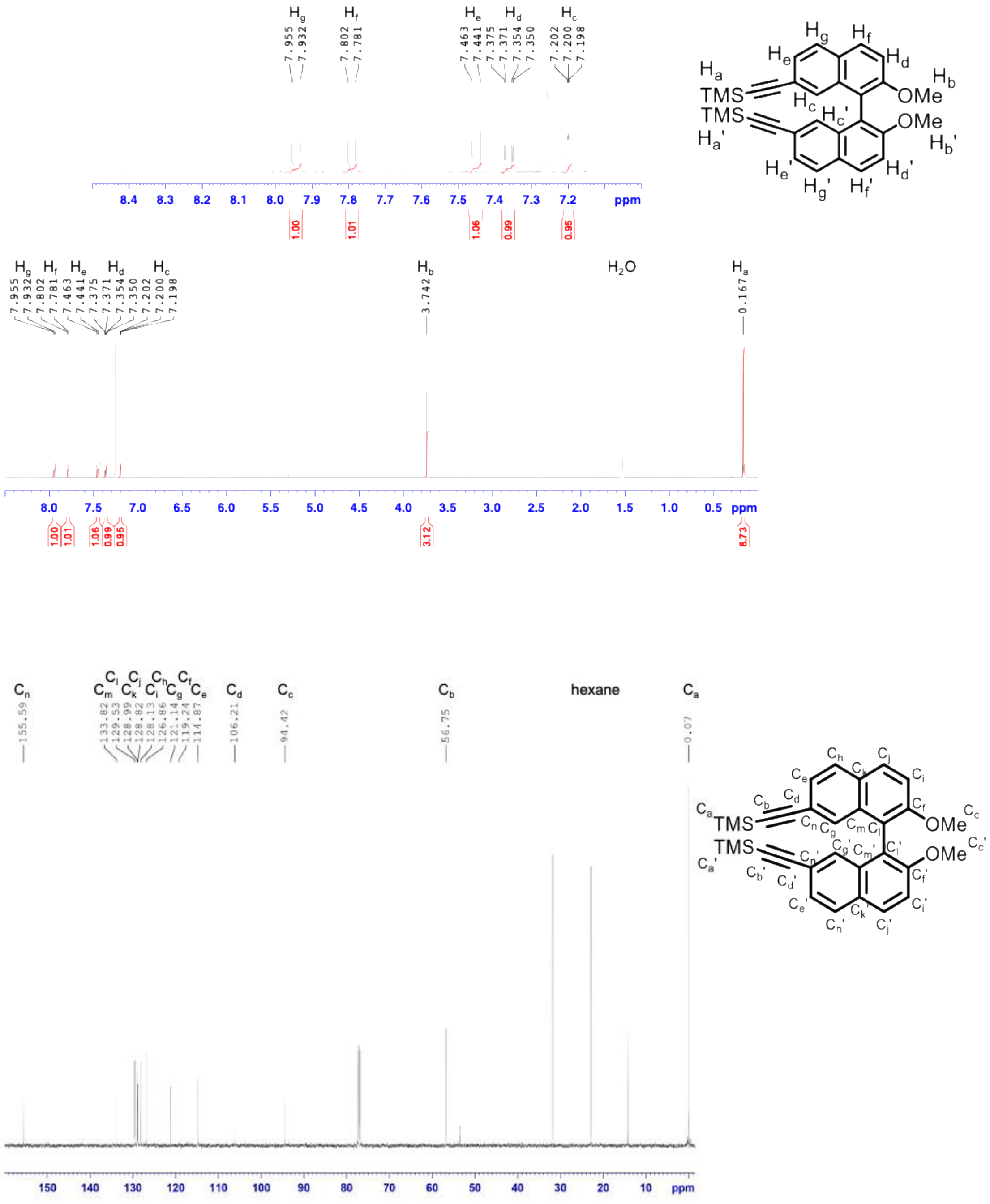

Figure S1: ^{1}H (top) and ^{13}C NMR spectra (bottom) of ***S*-2**

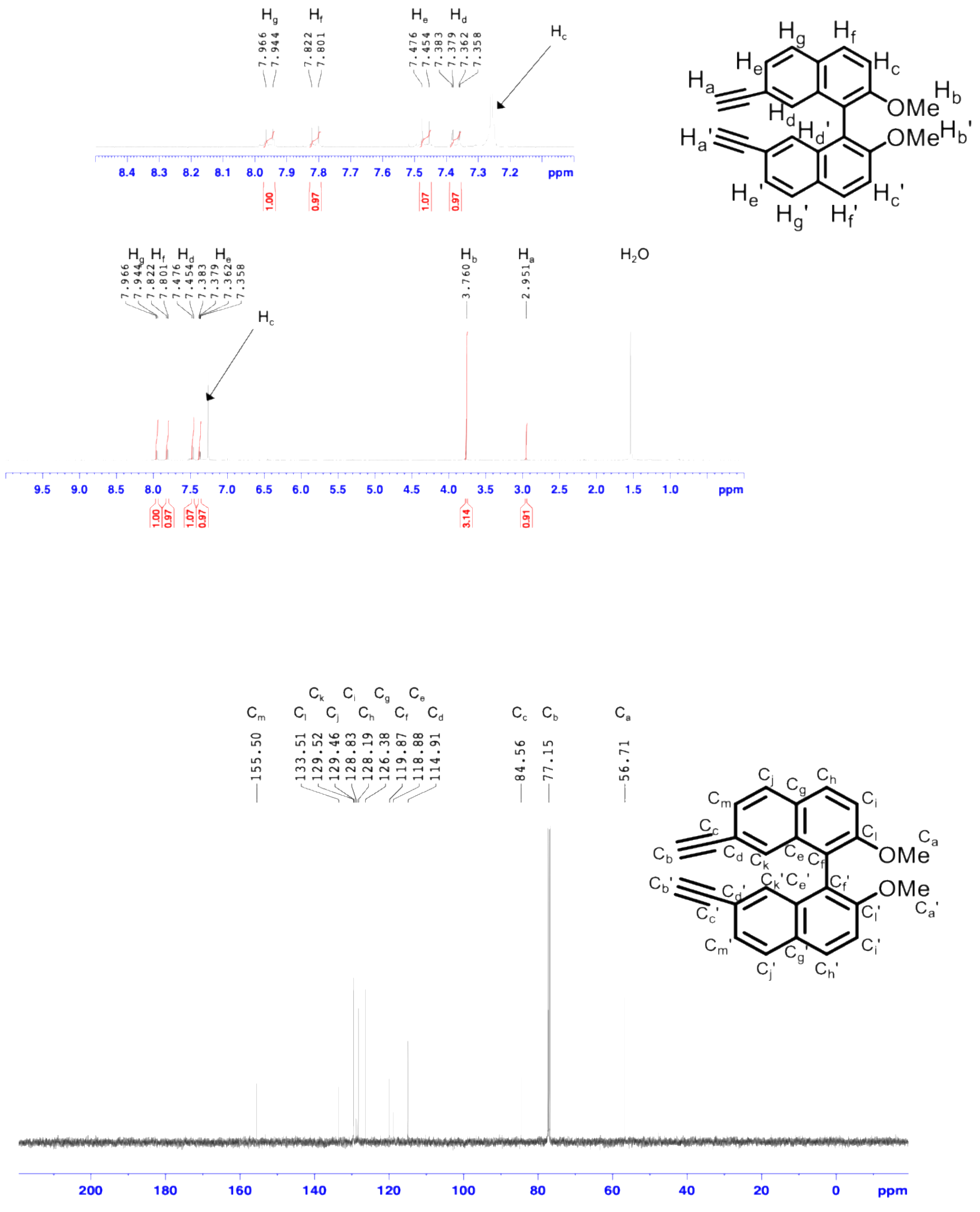

Figure S2: ^{1}H (top) and ^{13}C NMR spectra (bottom) of ***S*-3**

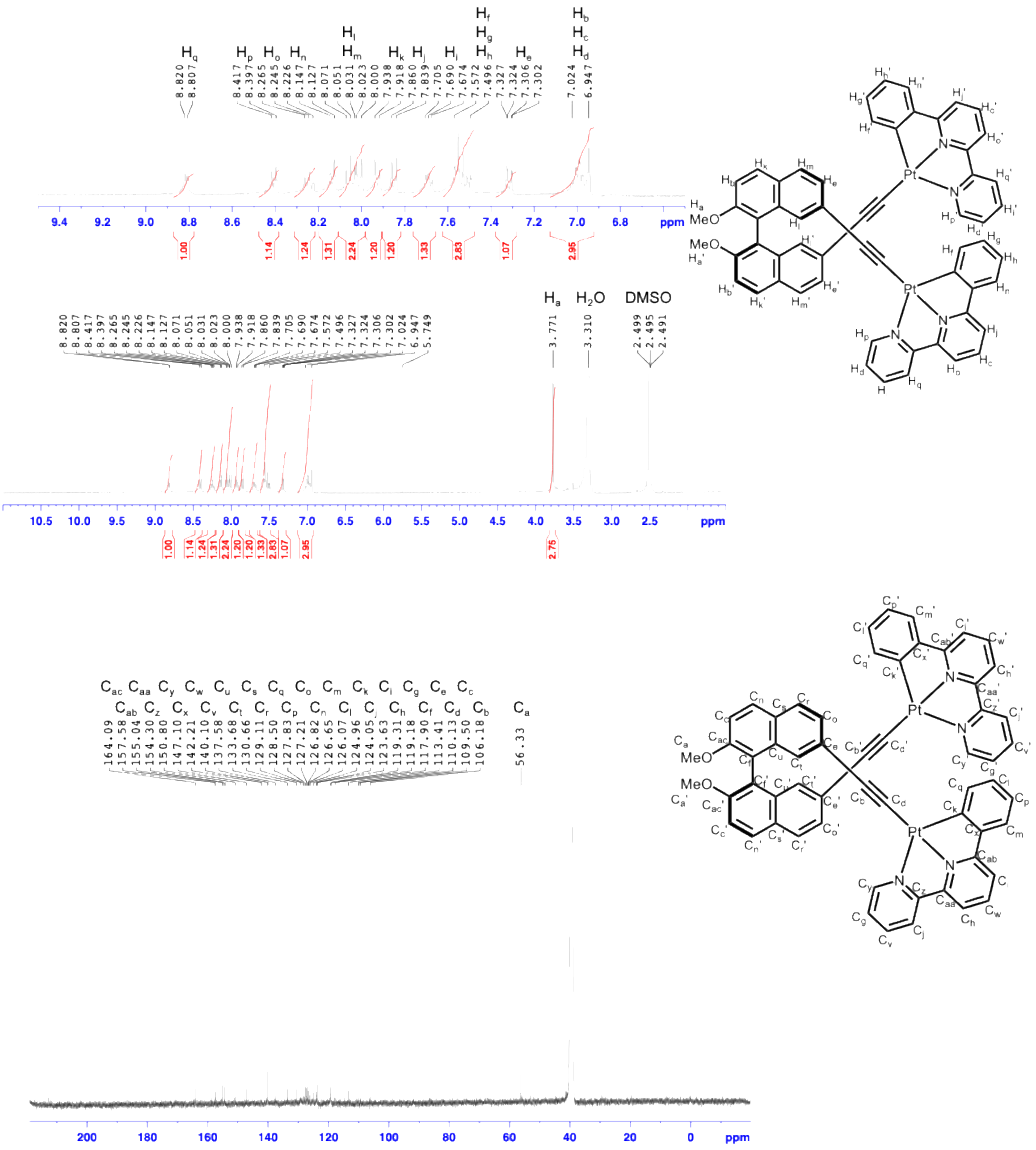

Figure S3: ^{1}H (top) and ^{13}C NMR spectra (bottom) of ***R*-Pt**

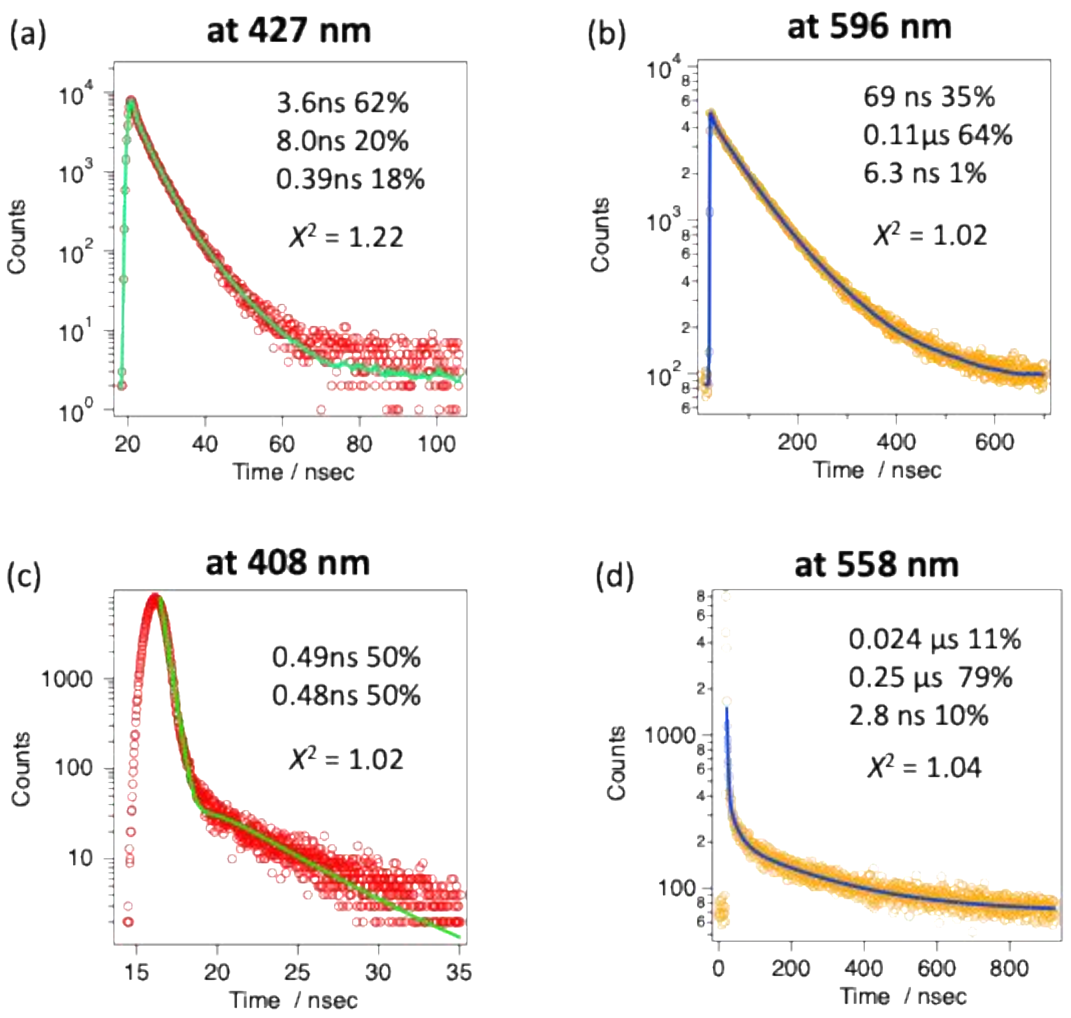

Figure S4: **(a)** Emission lifetime of dichloromethane solution at 427 nm, **(b)** at 596 nm (λ_{ex} = 370 nm). **(c)** Emission life time of PMMA matrix at 408 nm **(d)** at 558 nm.

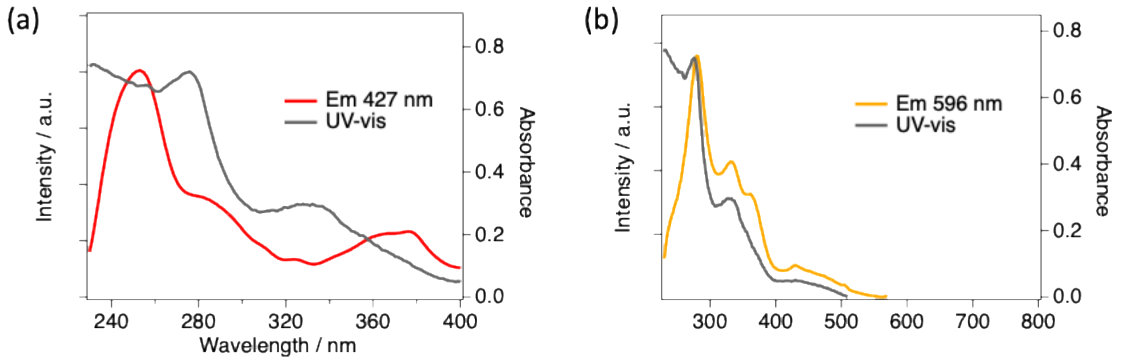

Figure S5: Excitation spectra and UV–vis spectra of a 1.0×10^{-5} M solution of ***R*-Pt** in dichloromethane: (a) at an emission wavelength of 427 nm, (b) at an emission wavelength of 596 nm.

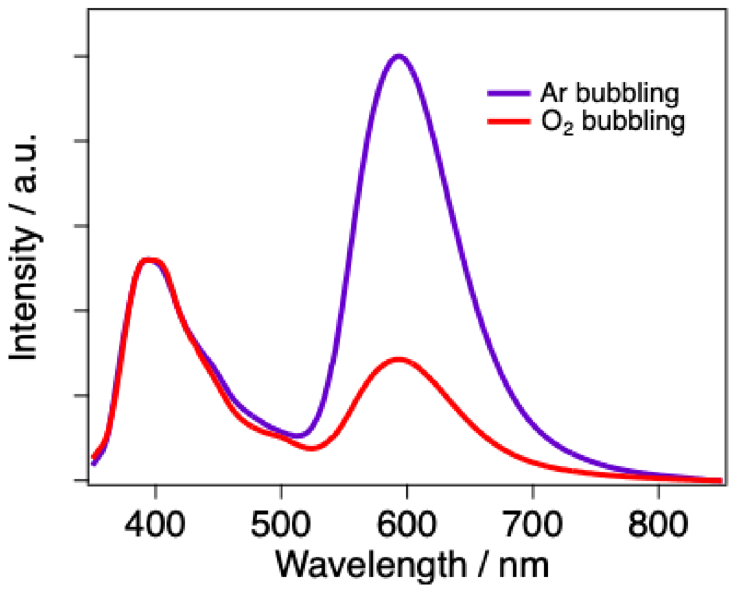

Figure S6: Emission spectra of a 1.0×10^{-5} M ***R*-Pt** solution in dichloromethane under Ar and O_2 bubbling conditions (λ_{ex} = 320 nm).

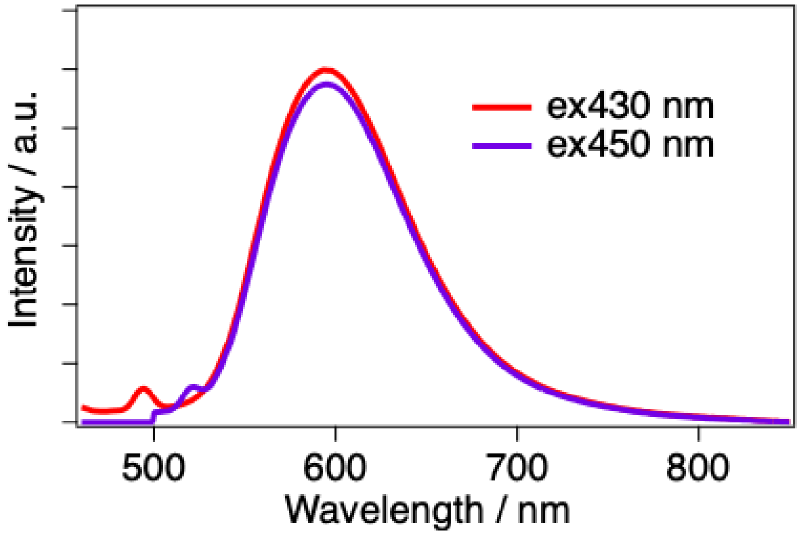

Figure S7: Emission spectra excited at the CT band of the absorption spectrum.

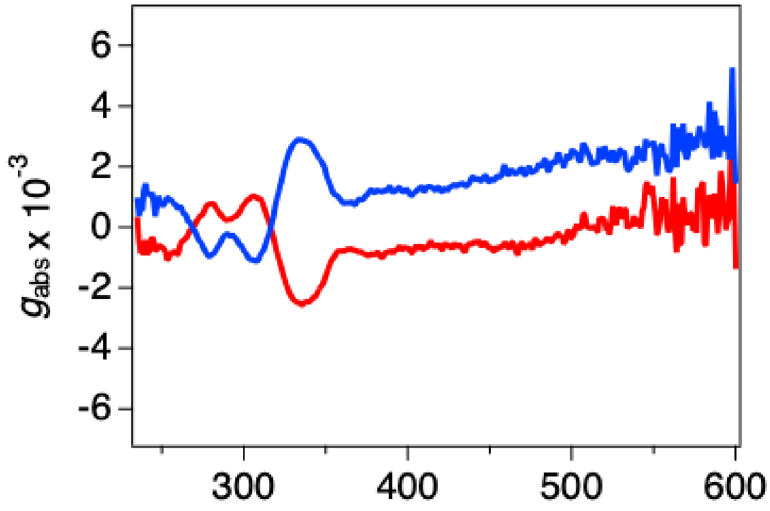

Figure S8: g_{abs} chart of ***R*/*S*-Pt** in 1.0×10^{-5} M dichloromethane solution.

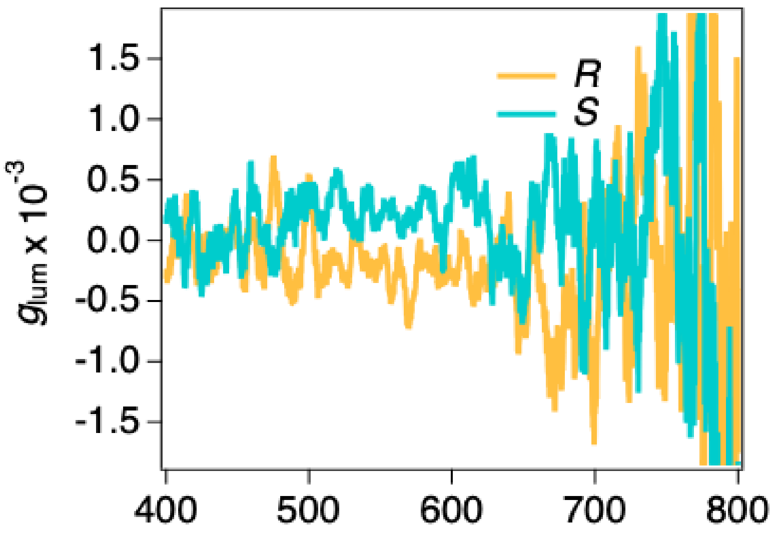

Figure S9: g_{lum} chart of ***R*/*S*-Pt** in PMMA matrices.

2. Theoretical calculations

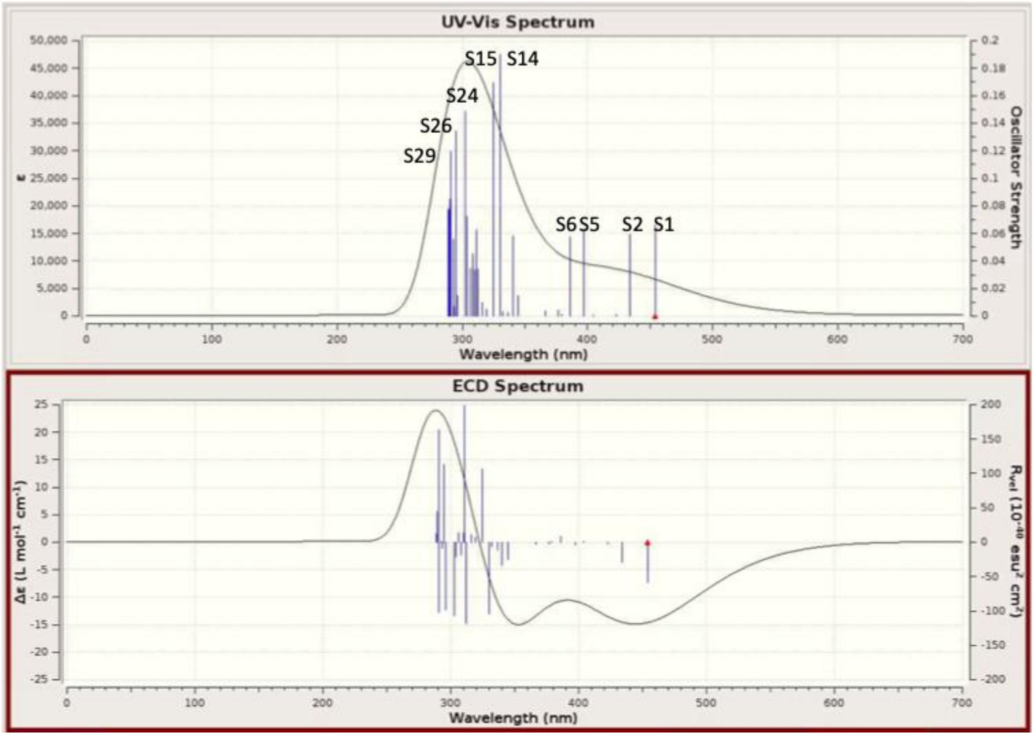

Figure S10: Simulated UV–vis and CD spectrum of ***R*-Pt** calculated by CAM-B3LYP/6-31+G(d,p).

Table S1: TD-DFT calculated excitation energies of ***R*-Pt** corresponding to the absorption wavelengths and main weight of transition.

Exited state	Excitation energy (OSC)	Dominant transitions (% weight)
**1**	2.7318 eV 453.86 nm (*f* = 0.0663)	232→234 (73%)
**2**	2.8576 eV 433.87 nm (*f* = 0.0594)	233→235 (85%)
**5**	3.1217 eV 397.17 nm (*f* = 0.0632)	227→234 (72%)
**6**	3.2125 eV 385.94 nm (*f* = 0.0578)	229→235 (100%)
**14**	3.7553 eV 330.16 nm (*f* = 0.1902)	226→236 (58%)
**15**	3.8173 eV 324.80 nm (*f* = 0.1698)	228→237 (82%)
**24**	4.1008 eV 302.34 nm (*f* = 0.1484)	221→234 (44%)
**26**	4.2097 eV 294.52 nm (*f* = 0.1345)	230→235 (21%)<br>231→241 (24%)
**29**	4.2622 eV 290.89 nm (*f* = 0.1196)	230→235 (22%)<br>232→240 (21%)

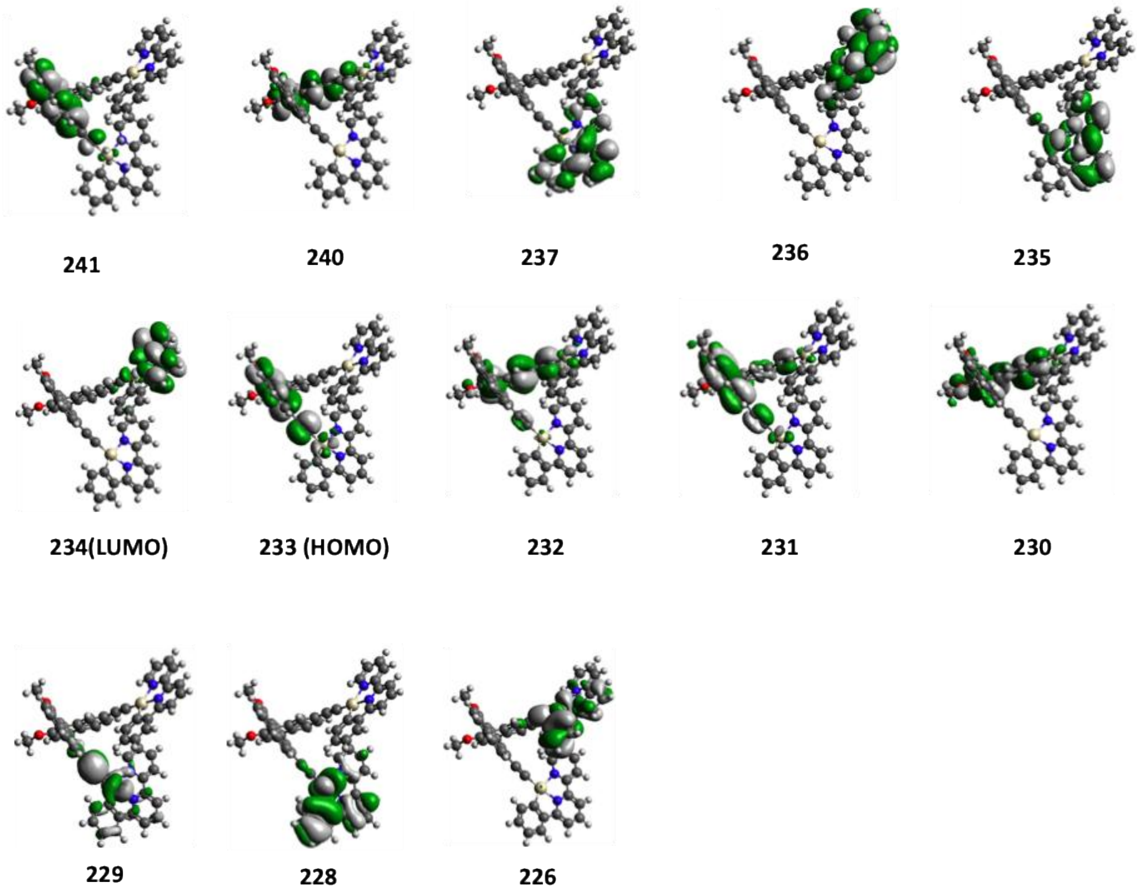

Figure S11: Dominant molecular orbitals of ***R*-Pt** related to the excitation listed in **Table S1.**

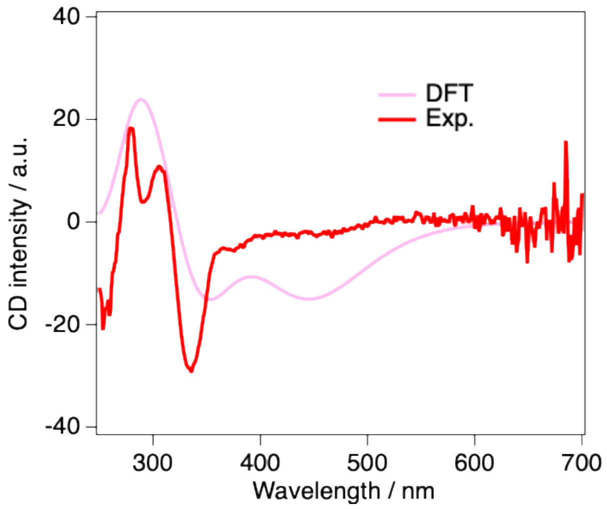

Figure S12: Experimental and simulated CD spectrum of ***R*-Pt** with TD-DFT calculation.

Table S2: Cartesian coordinates for ***R*-Pt** in ground state (S_0).

Atoms	X	Y	Z
C	1.07601	3.33509	-1.05702
C	0.23544	4.34734	-1.5934
C	0.04628	4.38624	-3.01675
C	0.71232	3.42887	-3.83099
C	1.50704	2.45612	-3.28267
H	1.22365	3.28458	0.01479
C	-0.43868	5.2973	-0.76205
C	-0.80536	5.36943	-3.56769
H	0.56848	3.47592	-4.90812
H	1.99954	1.72095	-3.91167
C	-1.462	6.27326	-2.76325
C	-1.28113	6.23562	-1.35809
H	-0.94786	5.40056	-4.64509
H	-2.11739	7.01046	-3.21097
C	-0.25259	5.32614	0.72211

C	-0.83961	4.34082	1.5791
C	0.49758	6.35522	1.28961
C	-1.58555	3.24319	1.07359
C	-0.65514	4.43755	3.00028
C	0.6727	6.44971	2.69235
H	-1.73232	3.15111	0.00435
C	-1.23091	3.44567	3.84202
C	0.10136	5.51032	3.5215
H	1.25576	7.25685	3.11896
C	-1.93239	2.38919	3.32285
H	-1.09232	3.53519	4.9171
H	0.23867	5.58414	4.59755
H	-2.35521	1.62886	3.97166
O	1.0505	7.25452	0.4181
O	-1.91455	7.10416	-0.51389
C	-2.79985	8.06674	-1.05454
H	-3.63848	7.60039	-1.58836
H	-3.19015	8.62521	-0.2017
H	-2.28736	8.76243	-1.7326
C	1.80995	8.33066	0.93381
H	2.69312	7.98424	1.48728
H	2.13982	8.90639	0.0668
H	1.21252	8.9803	1.58753
C	1.68911	2.38285	-1.8645
C	2.44845	1.31987	-1.28991
C	-2.10863	2.25994	1.90768
C	3.04196	0.38747	-0.75256
C	-2.76781	1.1176	1.36691
C	-3.27847	0.11557	0.87195
C	-8.29946	-2.42617	1.63152
C	-7.66826	-3.21484	0.67446
C	-6.37931	-2.88558	0.22781

C	-6.3573	-0.96554	1.70754
C	-7.64056	-1.30339	2.14418
H	-9.29714	-2.68242	1.9758
H	-8.18296	-4.0862	0.27692
H	-5.85838	-0.09156	2.11254
H	-8.13359	-0.68599	2.89126
C	-5.65934	-3.67654	-0.77992
C	-6.07618	-4.83321	-1.45337
C	-3.57683	-3.73368	-1.95032
C	-5.22168	-5.42795	-2.37959
H	-7.05281	-5.2592	-1.2556
C	-3.95991	-4.88645	-2.64001
H	-5.53887	-6.32328	-2.90568
H	-3.29973	-5.35343	-3.3608
C	-2.28102	-3.0196	-2.08381
C	-1.24782	-3.44008	-2.92376
C	-0.0592	-2.71135	-2.96746
H	-1.36625	-4.32976	-3.53178
C	-0.99514	-1.20008	-1.36705
C	0.07674	-1.57174	-2.17748
H	0.75291	-3.03589	-3.61083
H	-0.96022	-0.32279	-0.73039
H	0.98714	-0.98108	-2.16019
N	-2.13495	-1.90132	-1.3174
Pt	-3.88282	-1.50324	-0.06032
C	4.71558	-4.44658	2.61318
C	4.13196	-3.34139	1.97533
C	6.10862	-3.04833	0.66655
C	6.71986	-4.1438	1.27977
C	6.0044	-4.83819	2.26003
H	4.16661	-4.98925	3.37365
H	7.72151	-4.45423	1.00829

H	6.46007	-5.69278	2.75073
C	6.7052	-2.19041	-0.38889
C	7.99764	-2.37072	-0.88992
C	8.47809	-1.51227	-1.87611
H	8.62306	-3.17075	-0.51139
C	6.38036	-0.35762	-1.807
C	7.65843	-0.48633	-2.34482
H	9.48101	-1.6435	-2.27022
H	5.69103	0.41885	-2.12368
H	7.99609	0.20427	-3.10967
C	2.80347	-2.75538	2.18383
C	1.88115	-3.26235	3.11366
C	1.22897	-1.01384	1.57916
C	0.63844	-2.65561	3.26896
H	2.13016	-4.13164	3.71746
C	0.31549	-1.52928	2.50331
H	0.97143	-0.13174	1.00412
H	-0.07542	-3.05351	3.98387
H	-0.65395	-1.0509	2.61628
N	5.91533	-1.18391	-0.86002
Pt	3.95022	-1.11605	0.12901
N	-4.42776	-3.17866	-1.06186
C	-5.69381	-1.73816	0.74681
N	4.85914	-2.69096	1.02918
C	2.47817	-1.61161	1.38483
